# Rapid Indian Ocean warming fuels more frequent extreme pre-flood season rainfall over southern China

**DOI:** 10.1093/nsr/nwaf298

**Published:** 2025-07-23

**Authors:** Ruiqin Hou, Wenjun Zhang, Suqiong Hu, Rongrong Xu

**Affiliations:** CIC‐FEMD/KLME, State Key Laboratory of Climate System Prediction and Risk Management, Nanjing University of Information Science and Technology, Nanjing 210044, China; CIC‐FEMD/KLME, State Key Laboratory of Climate System Prediction and Risk Management, Nanjing University of Information Science and Technology, Nanjing 210044, China; Nicholas School of the Environment, Duke University, Durham, NC 27708, USA; CIC‐FEMD/KLME, State Key Laboratory of Climate System Prediction and Risk Management, Nanjing University of Information Science and Technology, Nanjing 210044, China

**Keywords:** pre-flood season rainfall, southern China, Indian Ocean warming, convection efficiency

## Abstract

In 2024, southern China faced its worst flooding during the pre-flood season (April–June), the first major rainfall season in East Asia, with considerable socioeconomic consequences. This extreme flooding is fueled by the unprecedented warming in the Indian Ocean, with a decaying moderate El Niño in the Pacific contributing weakly. Alarmingly, similar pre-flood season flooding events have become increasingly frequent in southern China over recent decades, posing unexpected risks to local communities. We demonstrate that the recent rapid Indian Ocean warming enhances local convection efficiency, leading to more frequent intense pre-flood season rainfall. As sea surface temperature in the Indian Ocean continues to rise in a warming world, it becomes increasingly crucial to understand its role in shaping regional extreme weather patterns for future climate adaptation and disaster management.

## Teaser

A fast-warming Indian Ocean is emerging as a key trigger for more frequent extreme pre-flood season rainfall in southern China.

## INTRODUCTION

The East Asian summer monsoon, typically spanning from late spring to early autumn, is essential for providing rainfall that supports billions of people [1–3]. Every few years, strong positive precipitation anomalies along the southwest-to-northeast oriented rainfall belt from the Yangtze River in China toward Japan during the plum rain season in boreal summer can lead to flooding in these regions [4–6]. Prior to the plum rain season, the monsoonal rainfall belt is positioned over southern China during late spring, marking the first major rainfall season in East Asia. This season is frequently linked to severe flooding events that have profound socioeconomic consequences.

**Figure 1.**
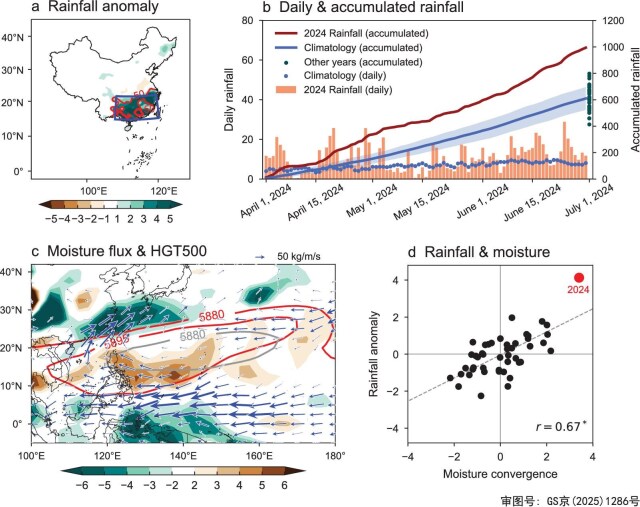
Record-breaking pre-flood season rainfall event over southern China in 2024. (a) Rainfall anomalies (shading; mm/day) during April–June 2024, with thin and thick red contours indicating anomalies exceeding 50% and 100% of the climatological mean, respectively. This plot uses the Lambert conformal conic projection. The blue box outlines the southern China region (22° –30°N, 105° –121°E) analyzed in this study. (b) Daily rainfall (bar; mm/day; left *y*-axis) and daily accumulated rainfall (red line; mm; right *y*-axis) over southern China from 1 April to 30 June 2024. Blue dots and the blue line represent climatological daily rainfall (mm/day; left *y*-axis) and climatological daily accumulated rainfall (mm; right *y*-axis), respectively. The blue shading indicates one standard deviation of the daily accumulated rainfall. Green dots represent accumulated rainfall (mm; right *y*-axis) for all other years during April–June from 1979 to 2024. (c) Anomalies of vertically integrated water vapor transport (vectors; kg/m/s), vertically integrated moisture divergence (shading; kg/m^2^/s). Red contours represent the 500 hPa geopotential height (HGT500, m) for April–June 2024, and the gray contour denotes the climatological 5880 m geopotential height line. (d) Scatterplot between vertically integrated moisture convergence and rainfall anomalies over southern China with a correlation coefficient (r) of 0.67. The year 2024 is marked by a red dot.

Over recent decades, an alarming increase in the frequency and intensity of pre-flood season flooding has been observed in southern China, particularly during the spring season of April to June [7–11]. The pre-flood season of 2022 witnessed unprecedented flooding across southern China, breaking historical records dating back to the 1960s. More recently, during April–June 2024, the region experienced its most severe flooding in four decades, with rainfall amounts surpassing those of 2022 by 25% (Fig. 1). This ‘once-in-a-century’ event resulted in catastrophic losses, including dozens of deaths and the evacuation of over 100 000 people. The growing frequency of such catastrophic events in pre-flood season poses unexpected risks to both human populations and vital infrastructure in one of the world's most densely populated and economically critical regions.

Concurrent with these extreme flooding events, significant sea surface temperature (SST) anomalies were observed across the Indo–Pacific Oceans. During the pre-flood season of 2024, coinciding with the decaying phase of a moderate El Niño event, SST warming occurred in the central tropical Pacific. It is well established that El Niño–Southern Oscillation (ENSO) exerts a substantial influence on pre-flood season rainfall over southern China by modulating regional air–sea interactions [12–15]. Specifically, during the spring of an El Niño decaying year, an enhanced anticyclone over the western North Pacific (WNP) facilitates increased moisture transport from tropical oceans into East Asia, leading to rainfall surpluses over southern China. Simultaneously, a pronounced Indian Ocean basin warming (IOBW) was detected, which is the dominant mode of SST variability in the Indian Ocean [16,17]. The IOBW typically develops in boreal winter, matures in spring, and persists into early summer [16,18,19]. The IOBW-related diabatic heating as a response to ENSO events can further affect rainfall patterns over East Asia by modulating both the intensity and location of the anomalous anticyclone over the WNP [16,19–21].

The climate impact of the Indian Ocean can sometimes extend beyond that of the ENSO, actively influencing regional climate around the globe rather than simply acting as an ENSO capacitor. Under the background of global warming, the Indian Ocean has undergone rapid warming over recent decades, at a rate faster than other tropical ocean basins [22,23]. This accelerated warming has increasingly amplified the role of the Indian Ocean SST variability in shaping global climate patterns [24–27]. As the Indian Ocean warms, it is expected to excite local atmospheric convection more efficiently, leading to increased latent heat release. This latent heating serves as a tropical heat source, driving stronger global atmospheric teleconnections [25]. It is compelling to consider that the ongoing Indian Ocean warming is actively contributing to more frequent pre-flood season flooding events over southern China. Understanding the mechanisms behind this increased frequency of the severe flooding is essential for improving predictive capabilities and developing effective mitigation strategies.

In this study, we first investigate the Indo-Pacific SST conditions associated with the extreme flooding event of 2024 over southern China, demonstrating a strong connection between this event and exceptional warming in the Indian Ocean. We then illustrate the significant role of the emerging Indian Ocean warming in the increasing frequency of severe pre-flood season flooding. Our findings indicate that since the 2000s, rapid warming of the Indian Ocean has sharply intensified its influence on pre-flood season rainfall over southern China. With further warming projected due to anthropogenic greenhouse gas forcing [28], the Indian Ocean is expected to play an increasingly critical role in shaping regional weather patterns and intensifying future flood risks.

## RESULTS

### Extreme pre-flood season rainfall in 2024 and its association with Indo-Pacific SSTs

We begin our investigation by analyzing the extreme pre-flood season flooding event over southern China during April–June 2024. Rainfall anomalies were concentrated over southern China (blue box in Fig. 1a), where substantial rainfall surpluses occurred, with maximum anomalies exceeding 5 mm/day (Fig. 1a). Most of the region experienced rainfall anomalies surpassing climatological levels by 50%. The temporal evolution of rainfall in 2024 showed that over two-thirds of the days in this season received surpluses relative to climatology (Fig. 1b). In particular, more than 30 days out of 3 months witnessed strong rainfall surpluses at least twice as large as the climatological mean. The extremity of this event is further evidenced by the daily accumulated rainfall (red line in Fig. 1b), which reached a total of 1000 mm, substantially exceeding the climatological mean of approximately 600 mm. This marked the wettest pre-flood season since 1979, surpassing the previous maximum by 25%.

This record-breaking flooding was concurrent with an intensification and westward shift of the western Pacific subtropical high, represented by the 500 hPa geopotential height (Fig. 1c). The enhanced subtropical high facilitated increased moisture transport into southern China from the tropical oceans along its northwest flank. The northeastward vertically integrated water vapor flux led to moisture convergence over southern China, contributing to abundant moisture accumulation there. As shown in Fig. 1d, there exhibits a good consistency between the integrated water vapor convergence and rainfall anomalies over southern China (r = 0.67, significant at the 95% confidence level). The extreme rainfall event of 2024 was associated with the strongest water vapor convergence recorded since 1979, suggesting the crucial role of moisture transport from tropical oceans.

**Figure 2.**
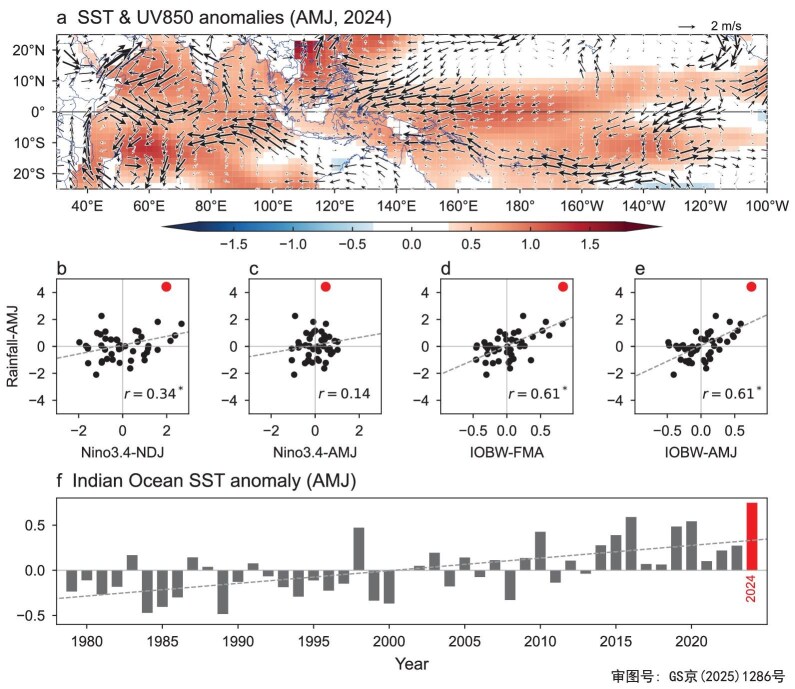
Relationship between tropical Indian Ocean SST and pre-flood season rainfall over southern China. (a) SST (shading; °C) and 850 hPa wind (vectors; m/s) anomalies during April–June (AMJ) 2024. Scatterplots of AMJ rainfall anomalies over southern China versus (b) the preceding winter (NDJ, December–February) Niño3.4 index, (c) the simultaneous (AMJ) Niño3.4 index, (d) the preceding spring (FMA, February–April) IOBW index, and (e) the simultaneous (AMJ) IOBW index. (f) Time series of the AMJ IOBW index from 1979 to 2024, with the year 2024 marked by a red bar. The dashed gray line represents the associated linear trend over this period.

To explore the potential drivers of this extreme event, we examined air–sea conditions in the tropical Indo-Pacific oceans (Fig. 2a). An evident SST warming appeared in the central Pacific, situating a decaying phase of a moderate El Niño event. A moderate positive correlation exists between pre-flood season rainfall anomalies and previous winter Niño3.4 index (r = 0.33, significant at the 95% confidence level) (Fig. 2b), consistent with previous studies [14,15]. In contrast, no significant relationship was found with simultaneous ENSO conditions (Fig. 2c), suggesting that the lagged ENSO influence on precipitation over China is possibly mediated through other ocean–atmosphere processes, such as the Indian Ocean capacitor effect [16,19]. This is supported by the regression of tropical Indo-Pacific SST anomalies onto the simultaneous April–June rainfall anomalies over southern China (Fig. S1).

Instead, the pre-flood seasonal rainfall anomalies are strongly correlated with the Indian Ocean basin SST anomalies. A line of studies have indicated that Indian Ocean warming can enhance the WNP anticyclone, leading to increased rainfall over East Asia [16,19]. The scatterplots in Fig. 2d and e demonstrate a strong relationship between the IOBW and pre-flood season rainfall, both during its peak phase (February–April) and late spring (April–June) (r = 0.61, significant at the 95% confidence level). The IOBW usually leads to more-than-normal rainfall during the pre-flood season by enhancing the moisture transport to southern China (Fig. S2). The positive relationship between the IOBW and pre-flood season rainfall remains robust after removing linear trends related to global warming (Fig. S3).

The IOBW is usually described as a response of local air–sea system to remote ENSO fluctuations [16,29]. Its variability is also significantly shaped by the rapid warming trend in response to anthropogenic forcing [30], which could potentially explain the record-breaking warming observed in late spring 2024 (Fig. 2f). This extreme Indian Ocean SST warming likely played a significant role in producing the largest rainfall surpluses over southern China during the pre-flood season (Fig. 2e). Indeed, four of the five extreme pre-flood season rainfall events since 1979 coincided with strong Indian Ocean warming, reinforcing the importance of IOBW in extreme flooding events.

### Increasing frequency of extreme pre-flood season flooding fueled by rapid Indian Ocean warming

**Figure 3.**
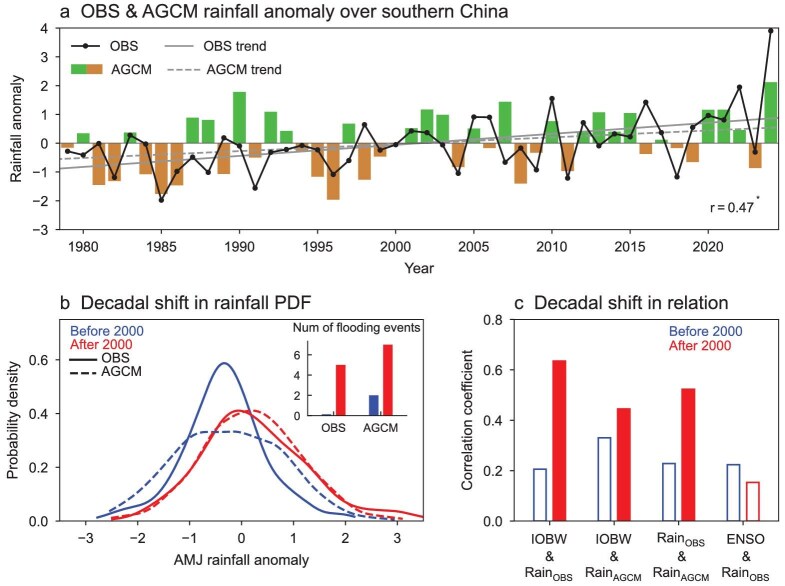
Decadal shift in observed and simulated pre-flood season rainfall features around 2000 and its connection with IOBW. (a) Time series of normalized pre-flood season rainfall anomalies over southern China in observations (OBS, black line) and atmospheric general circulation model (AGCM) simulations (bar). The solid and dashed gray lines denote the trends of observed and simulated rainfall anomalies, respectively. (b) PDFs of pre-flood season rainfall over southern China for the periods before 2000 (blue) and after 2000 (red). Solid lines represent observations, and dashed lines represent simulations. The inset indicates the number of flooding events in the observation and simulation. AMJ, April–June. (c) Correlation coefficients between observed IOBW and pre-flood season rainfall over southern China, observed IOBW and simulated pre-flood season rainfall over southern China, observed and simulated pre-flood season rainfall over southern China, and observed Niño3.4 index and pre-flood season rainfall over southern China.

Concurrent with the rapid Indian Ocean warming, the pre-flood season rainfall over southern China has exhibited an increasing trend, with more frequent strong rainfall events (solid black line in Fig. 3a). To understand their physical linkage, we conducted 15-member Atmospheric General Circulation Model experiments imposing observed monthly SST anomalies in the tropical Indian Ocean and climatological SST elsewhere (Fig. S3, see details in Methods). These simulations show that Indian Ocean SST anomalies can induce a significant increase in pre-flood season rainfall over southern China (dashed gray line in Fig. 3a). In particular, the record-breaking Indian Ocean warming in 2024 leads to the most severe rainfall event in model simulations. Over half of the rainfall anomaly in 2024 can be solely attributed to warming in the Indian Ocean. It is also noted that the simulated pre-flood season rainfall anomalies vary largely consistent with the observation since the 2000s, whereas such a connection is less apparent before (Fig. 3c).

We then roughly categorized the period into two intervals, using the year 2000 as a reference point, to examine their differences. First, we analyze the probability density functions (PDFs) to explore changes of rainfall characteristics. As shown in Fig. 3b, there is a clear rightward shift in the PDFs from the pre- to post-2000 periods with a more positively skewed structure, suggesting an increase in both the mean rainfall and the frequency of extreme events. When measuring extreme rainfall events as those exceeding one standard deviation, no such events were observed before 2000, whereas five pre-flood seasons reached this extreme level after 2000 (see inset in Fig. 3b). Similar shifts are detected in the simulations driven by Indian Ocean SST anomalies, though with a relatively weaker extension of the tail to the right. Correspondingly, the number of extreme rainfall events increases from two before 2000 to seven after 2000.

Next, we investigate the role of the tropical Indian Ocean SST in changes of pre-flood season rainfall around 2000. While no significant correlation is observed between the IOBW and pre-flood season rainfall anomalies before 2000, a strong relationship emerges after 2000, with the correlation coefficient reaching as high as 0.64 (statistically significant at the 95% confidence level) (Fig. 3c). This enhanced linkage between Indian Ocean SSTs and pre-flood season rainfall over southern China since the 2000s is also reproduced in our simulations, although the increase is weaker. As a result of this shift, the consistency between simulated and observed pre-flood season rainfall anomalies has improved significantly after 2000 (Fig. 3c). Note that the tropical Pacific SST anomalies do not exhibit any significant relationship with pre-flood season rainfall either before or after 2000, again suggesting minor roles of the tropical Pacific SST anomalies in driving the pre-flood season rainfall anomalies over southern China.

**Figure 4.**
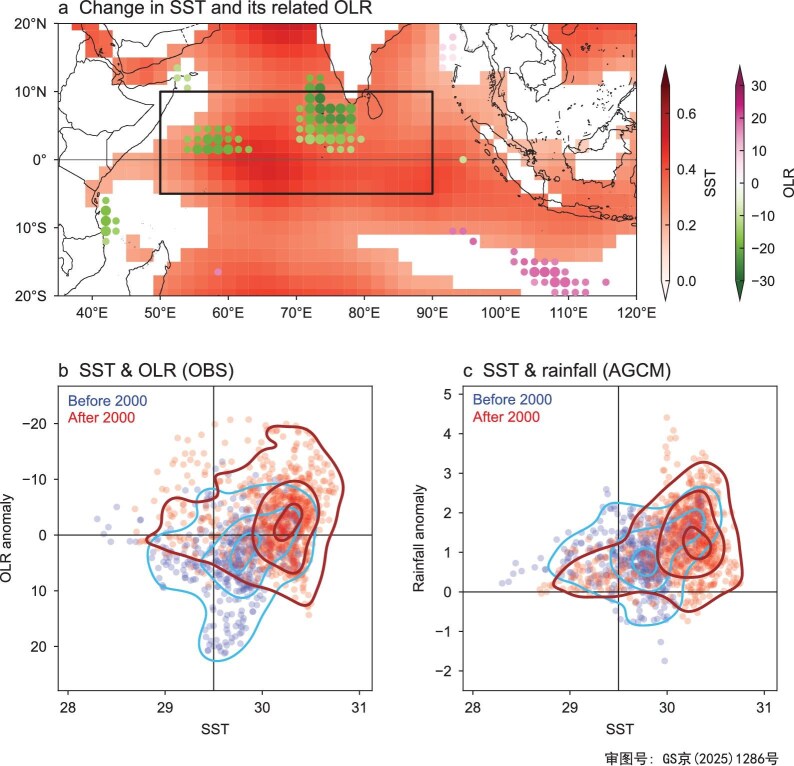
Enhanced SST–convection linkage around the year 2000. (a) Difference in IOBW regressed outgoing longwave radiation (OLR) (W/m^2^; small and large dots indicate statistical significance of the values at the 90% and 95% confidence level, respectively) and SST (°C; only showing values significant at the 95% confidence level) anomalies for April–June between the pre- and post-2000 periods. (b) Scatterplot of April–June OLR versus SST anomalies at each grid within 5°S–10°N, 50°–90°E [black box in (a)] for extreme IOBW events during the pre-2000 period (blue dots: 1983, 1987, 1998) and the post-2000 period (red dots: 2010, 2016, 2019, 2020, 2024). OBS, observed. (c) Same as (b), but for scatterplot of simulated rainfall and SST anomalies. AGCM, atmospheric general circulation model.

The influence of tropical SST on regional climate anomalies is known to be mediated by the excitation of local convection. Based on our finding of a significant relationship between the IOBW and southern China rainfall in the post-2000 period, we explore the local SST–convection linkage by linearly removing ENSO signals since the 2000s. This analysis reveals that enhanced convection appears primarily in the equatorial Indian Ocean (Fig. S4). Convection anomalies usually exhibit a non-linear response to the underlying SSTs, depending on both the background state and the superimposed anomalies [31,32]. Specifically, convection increases only weakly as SSTs rise up to the convection threshold, beyond which it intensifies rapidly. Consequently, similar SST anomalies, superimposed on different background states, can produce markedly different convection responses. Figure 4a illustrates the change in the local SST–convection relationship in the tropical Indian Ocean, with the removal of long-term trends to focus on their interannual relationship. The rapid warming of the Indian Ocean since 2000 has increased the sensitivity of local convection, as evidenced by a significant enhancement in SST-related convection anomalies over the northern off-equatorial region (Fig. 4a). This enhanced convection then favors a stronger response in the WNP circulation, contributing to more frequent intense pre-flood season rainfall over southern China.

Given this non-linear SST–convection linkage, strong SST warming is expected to induce larger convection anomalies during the warming period after the 2000s than during the cooler period before the 2000s. To further investigate the role of extreme IOBW events, we show the SST–convection relationship for the tropical Indian Ocean region during extreme IOBW events in pre- and post-2000 periods. These extreme IOBW events are defined as years with detrended April–June IOBW values exceeding one standard deviation (i.e. 1983, 1987, 1998, 2010, 2016, 2019, 2020, 2024). As shown in Fig. 4b, the positive SST anomalies for the tropical Indian Ocean region have been more effective in inducing convection responses after 2000 with the background warming. These findings are supported by model simulations (Fig. 4c), which demonstrate that strong local SST variability in a warming environment more effectively excites local convection. This thereby amplifies pre-flood season rainfall anomalies over southern China by modulating the WNP atmospheric circulation.

## CONCLUSION AND DISCUSSION

The present study demonstrates that the record-breaking pre-flood season rainfall event in 2024 is closely linked to the rapid warming of the Indian Ocean. The frequency of similar extreme pre-flood season flooding events has markedly increased since the 2000s, a trend that coincides with accelerated warming of the Indian Ocean. This warming has heightened the sensitivity of atmospheric convection to underlying SST anomalies, leading to more frequent and intense pre-flood season flooding over southern China through the enhancement of moisture transport. These findings underscore the growing role of the Indian Ocean in modulating the frequency and intensity of pre-flood season rainfall over southern China. The observed shift around 2000 indicates that extreme rainfall events, such as the one in 2024, are likely to become increasingly common as Indian Ocean SST continues to warm in the future.

One may argue that a regime shift around 2000 could potentially explain the observed changes in the relationship between the IOBW and pre-flood season rainfall anomalies. Indeed, the tropical Pacific experienced a notable regime shift characterized by more frequent central-Pacific-type El Niño events around this period. However, from the observational data, it remains challenging to clearly distinguish whether the strengthened IOBW–rainfall relationship is due to a regime shift in the tropical Pacific or directly driven by the local Indian Ocean warming. The experiments clearly reproduce the observed rainfall anomalies over southern China in the post-2000 period, distinctly differing from the pre-2000 period. It suggests that the enhanced rainfall anomalies are primarily driven by local warming in the Indian Ocean rather than by regime shifts in other regions such as the tropical Pacific.

The record-breaking rainfall event of 2024 serves as an alarming reminder of the increasing impact of Indian Ocean SST variability on regional climate. As Indian Ocean SST increasingly exerts control over climate patterns across the globe [33–35], the prediction accuracy of extreme weather events for its high-impact regions, including southern China, will depend heavily on improved monitoring and modeling of these SSTs. This study highlights the urgent need for further research into the evolving role of the Indian Ocean in regional and global climate dynamics. It is essential that climate models, both regional and global, incorporate these shifts to enhance forecast accuracy and support the development of effective adaptive strategies. Continued efforts to deepen our understanding of ocean–atmosphere interactions, particularly in the Indian Ocean, will be crucial for building climate resilience in the face of increasing extreme weather events. As the frequency and intensity of such events rise, driven by ongoing Indian Ocean warming, advancing our predictive capabilities will be crucial for mitigating their impacts on vulnerable regions.

## METHODS

### Observation and statistics

The rainfall dataset used in this study is sourced from the Climate Prediction Center (CPC) Unified Gauge-Based Analysis of Daily Precipitation [36] and CPC Merged Analysis of Precipitation [37]. The monthly SST dataset is obtained from the National Oceanic and Atmospheric Administration (NOAA) Extended Reconstructed SST analysis, version 5 (ERSSTv5) [38]. The monthly mean outgoing longwave radiation (OLR) data are provided by the NOAA [39]. For the monthly atmosphere data, we employ the National Centers for the Environmental Prediction-National Center for the Atmospheric Research (NCEP-NCAR) reanalysis [40]. The Niño3.4 index is used to measure the ENSO intensity, calculated as the average SST anomalies within the region bounded by 5°S–5°N and 120°–170°W. The IOBW index is quantified as the mean SST anomalies in the region of 20°S–20°N, 40°–110°E, based on the previous definition [19]. The analysis period extends from January 1979 to June 2024, with anomalies computed relative to the monthly mean climatology over the entire study period. The focus of this study is on the pre-flood season (April–June). Statistical significance is assessed using a two-tailed Student's *t*-test.

### Numerical experiments

To evaluate the impact of tropical Indian SST anomalies on extreme rainfall events during the pre-flood season, a set of numerical experiments were conducted based on the Geophysical Fluid Dynamics Laboratory (GFDL) Atmospheric Model version 2.1 (AM2.1) [41]. In these experiments, the observed monthly SST anomalies were superimposed on the climatological annual cycle of SSTs within the tropical Indian Ocean (15°S–15°N, 40°–120°E) (Fig. S5). Climatological SSTs in other ocean basins were maintained to isolate the effects of Indian Ocean warming. This set of experiment consists of an ensemble of 15 simulations with perturbed initial conditions, integrated from January 1979 to June 2024. The ensemble mean is utilized to minimize the effects of internal atmospheric variability, enhancing the robustness of our results.

## Supplementary Material

nwaf298_Supplemental_File

## Data Availability

The data used to reproduce the results of this paper are available at: https://psl.noaa.gov/data/gridded/data.noaa.ersst.v5.html; https://psl.noaa.gov/data/gridded/data.ncep.reanalysis2.pressure.html; https://psl.noaa.gov/data/gridded/data.cpc.globalprecip.html; https://psl.noaa.gov/data/gridded/data.cmap.html; and https://psl.noaa.gov/data/gridded/data.interp_OLR.html. Codes and requests for materials used in this study are available from the corresponding author on request.
