# Supplementary material for: Rapid Indian Ocean warming fuels more frequent extreme pre-flood season rainfall over southern China
Source: Natl Sci Rev. 2025 Jul 23;12(10):nwaf298. doi: 10.1093/nsr/nwaf298 (PMC12418954; doi:10.1093/nsr/nwaf298)
Supplement: nwaf298_Supplemental_File [file nwaf298_supplemental_file.docx]

**Supplementary Information for:**

**Rapid Indian Ocean warming fuels more frequent extreme pre-flood season rainfall over southern China**

**Ruiqin Hou^1^, Wenjun Zhang^1*^, Suqiong Hu^2^, Rongrong Xu^1^**

^1^1CIC‐FEMD/KLME, State Key Laboratory of Climate System Prediction and Risk Management, Nanjing University of Information Science and Technology, Nanjing 210044, China

^2^Nicholas School of the Environment, Duke University, Durham, NC 27708, USA

^#^Corresponding author: Wenjun Zhang (zhangwj@nuist.edu.cn)


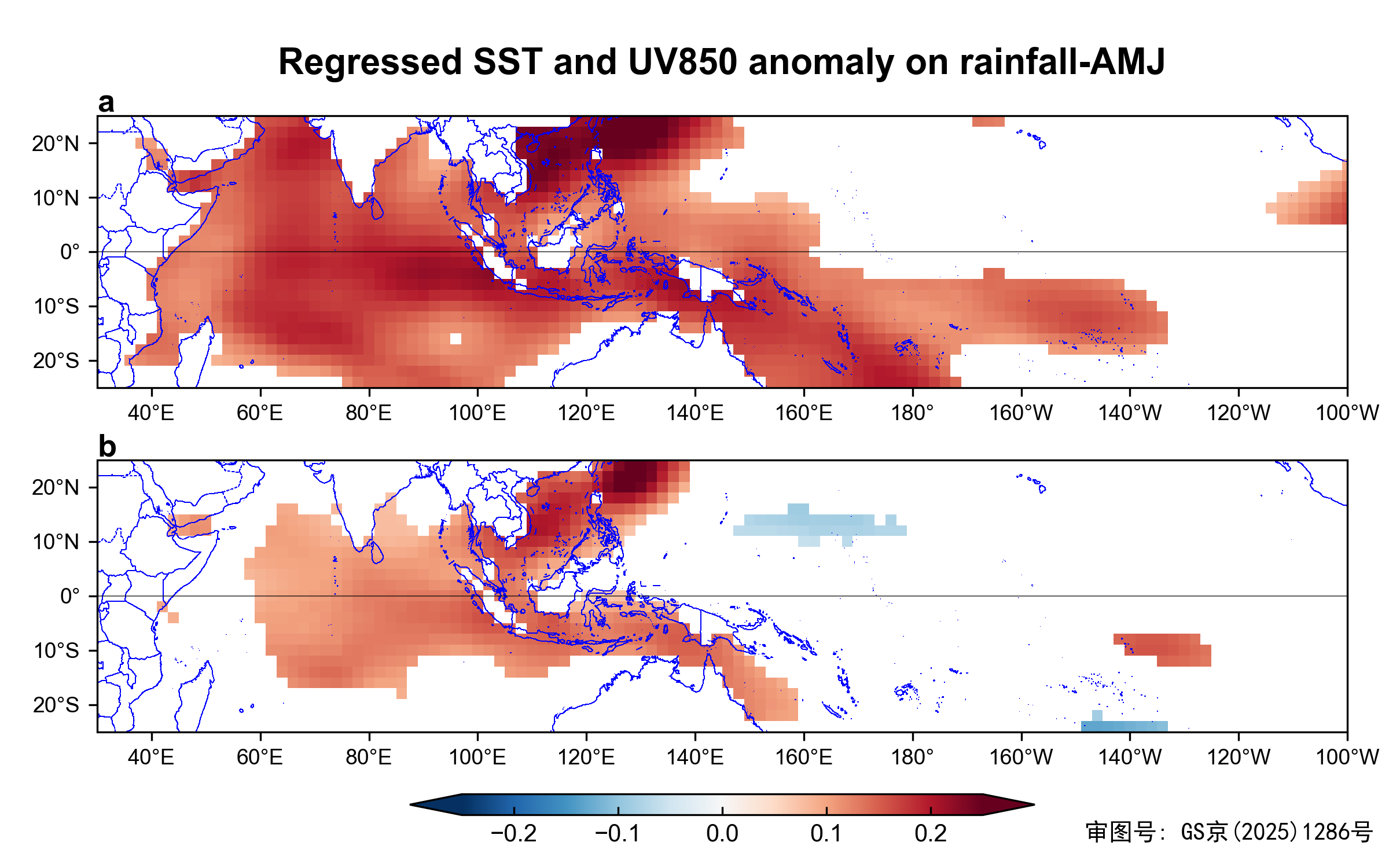


**Figure S1.** Regressed SST anomalies upon pre-flood season rainfall over southern China. **a** SST anomalies with the linear trend included and **b** SST anomalies with the linear trend removed. Shading represents SST anomalies that are statistically significant at the 90% confidence level.


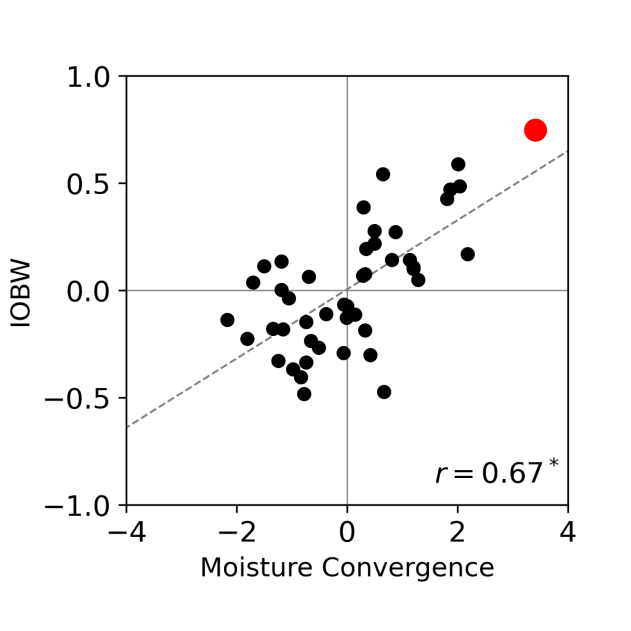


**Figure S2.** Scatterplot of IOBW versus vertically integrated moisture convergence over southern China with the correlation coefficient (r) of 0.67. The year 2024 is highlighted by a red dot.


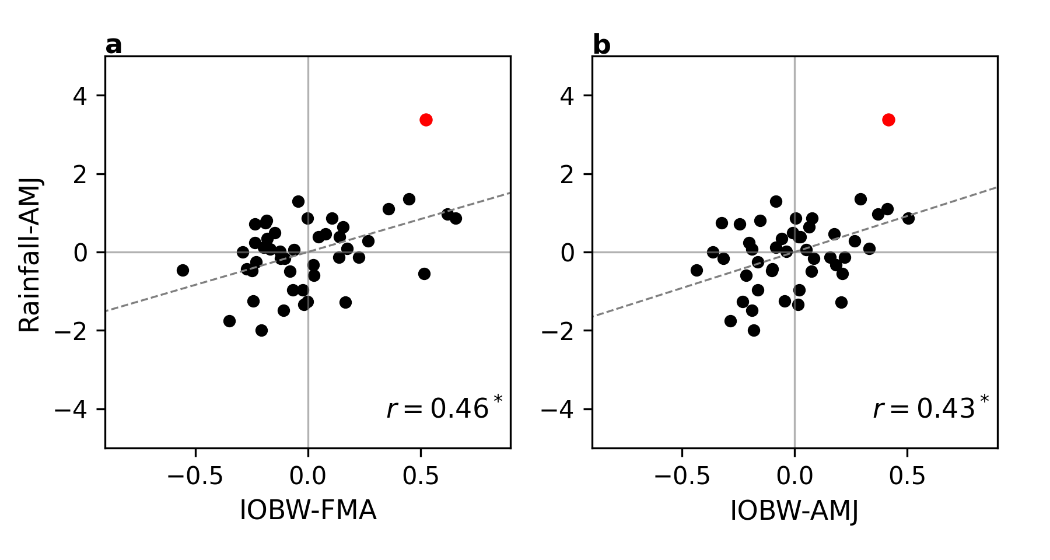


**Figure S3.** Scatterplots of April-June rainfall anomalies over southern China versus **a** the preceding spring (February-April) IOBW index and **b** the simultaneous (April–June) IOBW index, with respective linear trends removed.


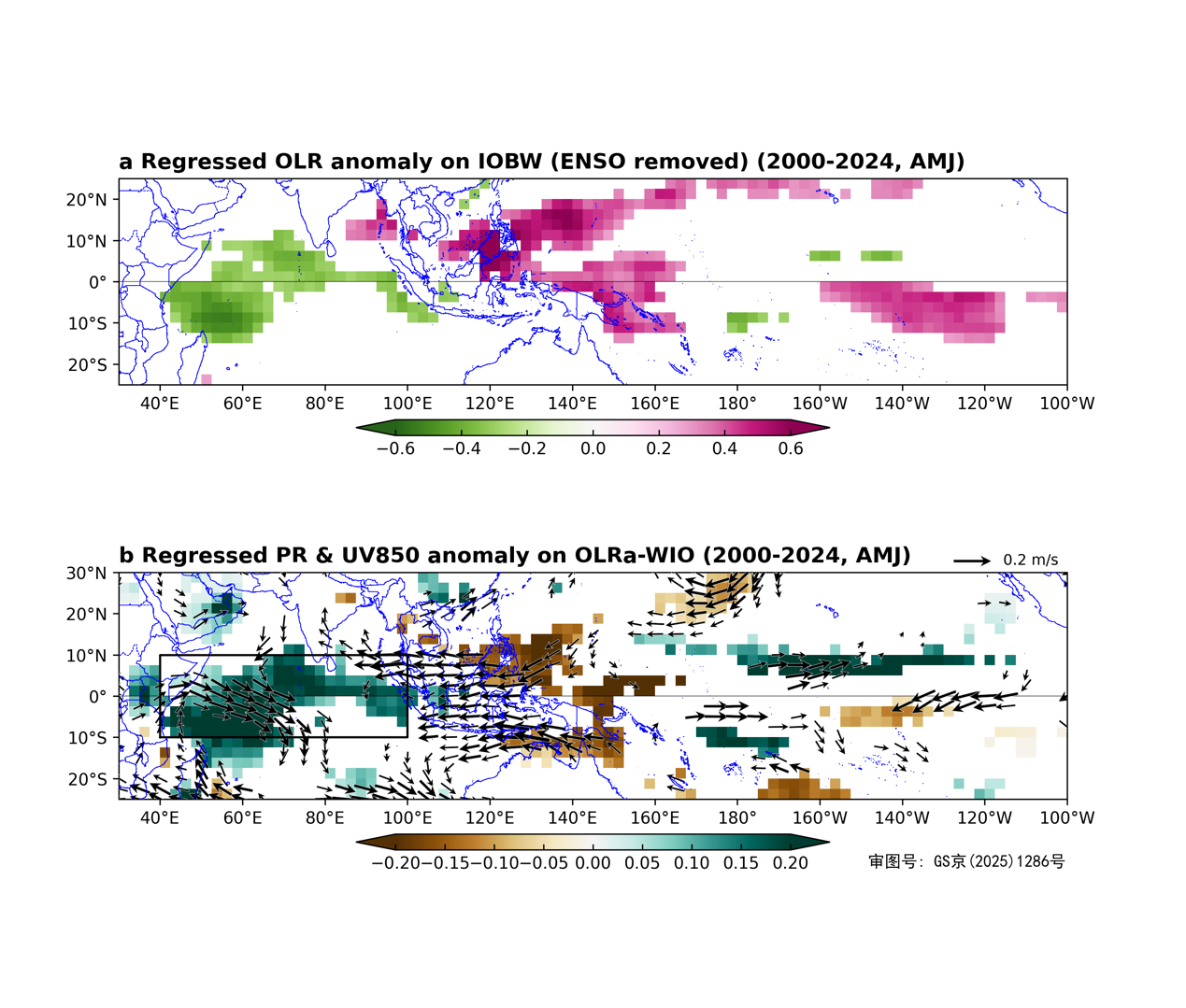


**Figure S4**. **a** Regression of convection anomalies (represented by OLR anomalies) onto the IOBW index during the pre-flood season for the post-2000 period, after linearly removing ENSO signals. **b** Regression of OLR and atmospheric circulation anomalies onto the convection index averaged over the equatorial Indian Ocean (region outlined by the black box).


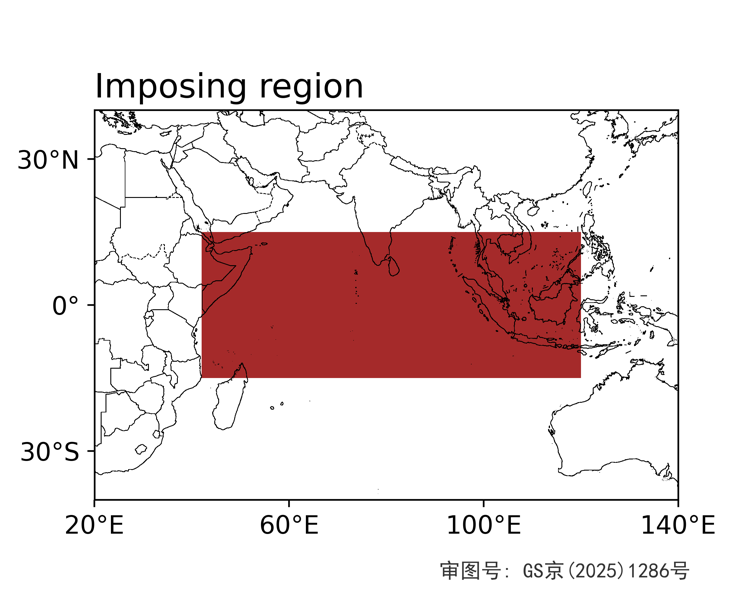


**Figure S5.** The region (15°S–15°N, 40°E–120°E) where the observed monthly SST anomalies are imposed in the AGCM experiments.
